# Bovine Corpus Luteum Proteomics during Different Reproductive and Physiological Stages

**DOI:** 10.1038/s41597-026-07515-6

**Published:** 2026-06-03

**Authors:** Granit Thaqi, Dapi Menglin Chiang, Susanne I. Wudy, Christina Ludwig, Bajram Berisha, Michael W. Pfaffl

**Affiliations:** 1 Chair of Animal Physiology and Immunology, School of Life Sciences, Technical University of Munich, Weihenstephan, Freising, München, Germany; 2 Bavarian Center for Biomolecular Mass Spectrometry (BayBioMS), School of Life Sciences, Technical University of Munich, Freising, München, Germany; 3 Department of Animal Biotechnology, Faculty of Agriculture and Veterinary, University of Prishtina, Prishtina, Kosovo

## Abstract

Corpus luteum (CL) is an endocrine structure that undergoes substantial changes over short intervals during the bovine reproductive (estrous) cycle. These changes are regulated by a vast array of signaling molecules, mainly proteins, that govern reproduction. We collected a comprehensive set of CLs from non-pregnant (days 1–2, 3–4, 5–7, 8–12, 13–17, and >18) and pregnant (months 1–2, 3–4, 5–6, and >7) cows, enabling a detailed characterization of temporal changes in CL biology. While previous studies have focused on a limited number of proteins, we employed a broader approach to exploring additional proteins potentially involved in CL regulation across different stages. Using high-resolution mass spectrometry (HR-LC-MS/MS), we identified approximately 3,783 individual proteins. These proteins may be useful for research in species where access to well-characterized CL tissue is limited. Our findings indicate diverse changes in protein expression across groups, highlighting proteomic patterns that may reflect key processes of bovine reproduction, particularly during the transition toward regression, a critical period that influences whether the CL is maintained to support pregnancy or undergoes regression.

## Background & Summary

The corpus luteum (CL) is a temporary but indispensable endocrine organ in all mammalian species, including domestic animals. Emerging from the remnants of the ovulated follicle, the CL plays a central role in orchestrating the estrous or menstrual cycle and in establishing pregnancy. Its primary function is the synthesis and secretion of progesterone, a steroid hormone that is essential for preparing the endometrium for embryo implantation, maintaining uterine quiescence, and regulating maternal responses to permit embryo survival. In contrast, estradiol, produced by the developing follicles, promotes endometrial proliferation and creates the optimal environment for follicular growth and ovulation. If pregnancy is not achieved, the CL undergoes regression (luteolysis), allowing a new reproductive cycle to begin. The beginning of a cycle is characterized by a decrease in both progesterone and estradiol levels following luteolysis. Despite its short lifespan, CL undergoes some of the most rapid and dramatic changes in structure, cells, and molecular activity in the body. These processes, which include its formation (luteinization), functional period, and luteolysis, make the CL an excellent model for studying tissue remodeling, endocrine regulation, and reproductive physiology^1,2^.

In domestic species such as cattle, pigs, sheep, and goats, CL function is a key determinant of reproductive success and therefore of great economic importance in agriculture worldwide^3–5^. The quality, duration, and endocrine output of the CL can influence pregnancy rates, embryonic survival, and the effectiveness of reproductive technologies like artificial insemination and embryo transfer^6^. This has motivated a growing number of studies focused on characterizing the luteal proteome across species and stages of the luteal phase. These studies have confirmed the critical roles of proteins involved in steroidogenesis (STAR, CYP11A1, HSD3B), lipid metabolism, oxidative phosphorylation, cytoskeletal remodeling, and cell signaling^7–11^.

Recent advances in molecular biology have highlighted the limitations of traditional genomics and transcriptomics in fully explaining CL function. While these methods provide information about gene expression and potential protein coding, they do not reflect the actual entire protein landscape and their connected pathways active at a given time^12^. Proteomics, which involves the large-scale identification and quantification of proteins, offers a more functional understanding of all cellular and reproduction important processes. It provides insight into not only the presence of proteins but also their abundance, post-translational modifications, interactions, dynamic and/or synergistic regulations. In the context of CL biology, proteomics has become an essential tool for deciphering the protein networks that drive its rapid formation, hormone output, and eventual regression. Furthermore, the CL is also a highly vascularized organ, requiring rapid blood vessel formation within days of ovulation^13^. Past proteomic investigations have consistently identified a broad array of angiogenic factors, including VEGF and matrix metalloproteinases, as well as components of the extracellular matrix like integrins and collagens^14–17^. These proteins support the rapid development of the luteal vasculature, which is necessary for delivering cholesterol precursors, removing metabolic waste, and transporting hormones systemically.

In the absence of pregnancy, the CL undergoes luteolysis, characterized by reduced steroidogenic capacity and increased inflammatory and apoptotic activity. Molecular studies have reported increased expression of factors associated with pro-inflammatory cytokines, oxidative stress responses, caspase-mediated apoptosis, and anti-angiogenic processes during this phase^14,18–20^. In contrast, pregnancy recognition signals in ruminants promote luteal maintenance by supporting cell survival and sustained progesterone production.

In this study we analysed protein expression in the bovine CL across different stages of the estrous cycle (days 1–2, 3–4, 5–7, 8–12, 13–17, and >18) and of pregnancy (months 1–2, 3–4, 5–6, and >7). Understanding proteomic changes across the lifespan of the CL is critical, as this dynamic structure undergoes formation, development, maintenance, and regression. We identified 3,783 proteins expressed across all groups, with differences between time points. This dataset provides a comprehensive proteomic profile of bovine CL and serves as an important resource to advance future investigations into the molecular dynamics of the ovary and CL.

Although not focused on individual protein regulation, our holistic high-resolution proteome analysis advances knowledge in reproductive biology. These findings may also reveal novel molecular networks, with bovine CL serving as a valuable model for species where well-defined CL tissue is less accessible.

## Methods

### Animals

The CL was collected from dairy cows (German Fleckvieh), from the local slaughterhouses, first frozen in liquid nitrogen, and then frozen at −80 °C until further processing for protein isolation techniques^15^. No animals were harmed or sacrificed specifically for this study. Samples were obtained from animals slaughtered for commercial purposes. Therefore, approval from the Institutional Animal Ethics Committee was not required.

### Female reproductive tract examination and corpus luteum collection

**Fig. 1:**
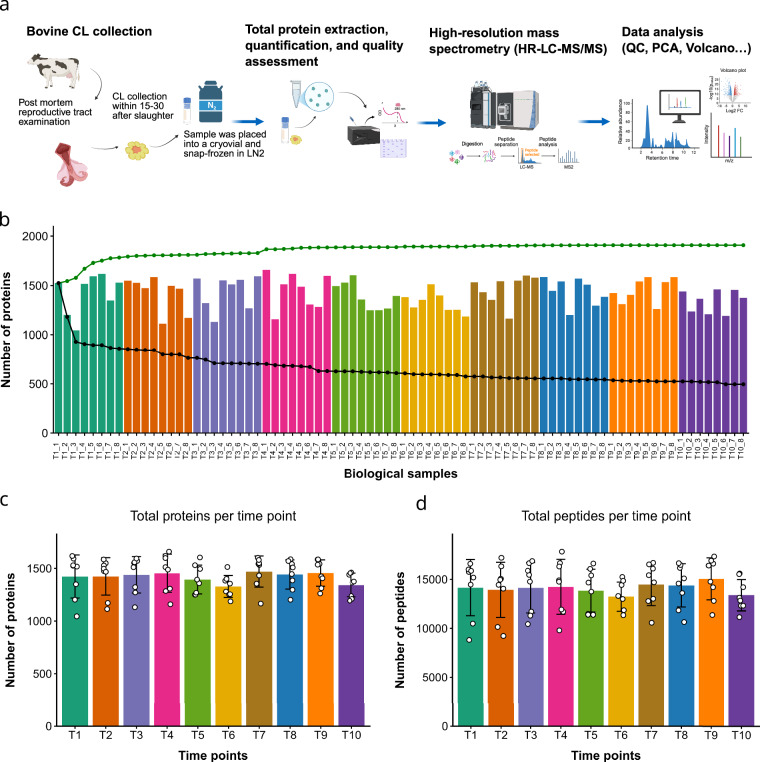
Schematic overview of the experimental and computational workflow used for the proteomics analysis. (**a**) Sample obtaining and preparation, protein extraction, mass spectrometry acquisition, and data processing. Created in BioRender. Thaqi, G. (2026) https://BioRender.com/ecinj1q. (**b**) Number of unique proteins identified across all samples, green trendline depicts cumulative proteins across all ten groups (T1–T10), as well with the black trendline as the subset of proteins consistently detected, illustrating both proteome depth and overlap. (**c**) Total number of proteins identified by timepoint group, providing an overview of detection robustness and variability across the dataset. (**d**) Consistency of peptide identification, represented by the distribution of peptide counts grouped by time point, highlighting reproducibility across the experimental timeline.

Cl samples were obtained during our previous project (Berisha *et al*.)^21^ and continuously expanded upon subsequent work (Thaqi *et al*.)^22^. Throughout the process, we always made sure that the samples were aliquoted and snap-frozen, which provides excellent protein integrity for MS-based proteomic profiling^23^. After post mortem macroscopic observation of whole reproductive tract, including ovaries, uterus, follicles (size, shape), corpus luteum (size, color, cavities, vacuoles)^24,25^, Cl was classified into the following stages of the estrous cycle: T1 (Day 1–2, n = 8), T2 (Day 3–4, n = 8), T3 (Day 5–7, n = 8), T4 (Day 8–12, n = 8), T5 (Day 13–17, n = 8), T6 (Day > 18, n = 8)^22,24^. In addition, in pregnant cows, we measured crown–rump length, evaluated placental characteristics (cotyledons, thickness), and examined fetal weight and organ development in pregnant cows four groups of pregnancy stages were classified: T7 (Month 1 and 2 of pregnancy, n = 8), T8 (Month 3–4, n = 8), T9 (Month 5–6, n = 8), T10 (Month > 7, n = 8)^22,26^. The CL samples were collected within 15-30 minutes of slaughter, minimizing cold ischemia time. The samples were divided and aliquoted, placed into sterile cryovials, and snap-frozen in liquid nitrogen (Fig. 1a). The vials were then transported within 1 hour to a −80 °C freezer at our Cl tissue bank for long-term storage. The aliquoted tissue was quickly cut into 40 mg portions at cool temperatures, avoiding thawing, and then processed for protein extraction.

### Protein extraction and peptide preparation

Total protein was extracted from 40 mg of frozen CL tissue, which had been precooled in liquid nitrogen for at least 1 minute. The frozen tissue was homogenized in a silica bead tube and subsequently transferred into lysis buffer containing 8 M urea, 4% CHAPS, 50 mM DTT, and protease inhibitor (cOmplete-ULTRA, Roche, Switzerland, 1 tablet per 10 mL extraction buffer). The lysate was centrifuged at 15,000 x g for 15 minutes at 4°C to remove cellular debris, and the supernatant was collected^15^. The supernatant was then subjected to dialysis using a membrane with a molecular weight cut-off (MWCO) of 12,000–14,000 Da (SERVAPOR^®^, Serva, Germany) in PBS at 4 °C for 24 hours to remove contaminants which may interfere later with the mass spectrometry analysis^27^. Protein concentration was determined using the Bradford assay. Total protein samples were stored at −80 °C until proteomic analysis (Fig. 1a). Peptide preparation was performed by in-solution digestion^28^. A total of 25 µg protein per sample was used for further processing. Disulfide bridges were reduced with 10 mM DTT at 30 °C for 30 min. Alkylation was performed at room temperature in the dark using 55 mM chloroacetamide for 30 min. Digestion was performed in two steps with trypsin (Trypsin Gold, Promega), each step at a 1:100 enzyme-to-substrate ratio and incubation for 2 hours (first step) and 18 hours (second step) at 30 °C. Digests were acidified by addition of formic acid (FA) to 1%, and desalted using self-packed StageTips (three disks, ø 1.5 mm, C18 material, 3 M Empore per micro-column). Eluates were dried down and stored at −80 °C.

### Proteomic data acquisition by mass spectrometry

LC-MS/MS data acquisition was carried out on a Dionex Ultimate 3000 RSLCnano system coupled to an Orbitrap Fusion LUMOS mass spectrometer (ThermoFisher Scientific, Bremen). 0.2 µg of total peptide amount per sample was delivered to a trap column (ReproSil-pur C18-AQ, 5 μm, Dr. Maisch, 20 mm × 75 μm, self-packed) at a flow rate of 5 μL/min in 0.1% formic acid in HPLC grade water. After 10 minutes of loading, peptides were transferred to an analytical column (ReproSil Gold C18-AQ, 3 μm, Dr. Maisch, 450 mm × 75 μm, self-packed) for one minute, and then separated using a 50 min gradient from 4% to 32% of solvent B (0.1% FA, 5% DMSO in acetonitrile) in solvent A (0.1% FA, 5% DMSO in HPLC grade water) at 300 nL/min flow rate. The Orbitrap Fusion LUMOS mass spectrometer was operated in data-dependent acquisition (DDA) and positive ionization mode. MS1 spectra (360–1300 m/z) were recorded at a resolution of 60k using an automatic gain control (AGC) target value of 4e5 and a maximum injection time (maxIT) of 50 ms. A cycle time of 2 s was set. Only precursors with charge states 2 to 6 were selected, and dynamic exclusion of 30 s was enabled. Peptide fragmentation was performed using higher energy collision-induced dissociation (HCD) and a normalized collision energy (NCE) of 30%. The precursor isolation window width was set to 1.3 m/z. MS2 Resolution was 15k with an AGC target value of 7.5e4 and maximum injection time (maxIT) of 22 ms. All proteomic samples were analyzed in randomized order, with a blank injection performed between each sample to allow column re-equilibration and to minimize protein carryover. Overall system performance was continuously monitored by regular injections of a quality control sample (Pierce HeLa digest).

### Peptide identification and quantification of MS data

Peptide identification and quantification were performed using the MaxQuant software (version 1.6.3.4)^29^, with its built-in search engine Andromeda^30^. MS2 spectra were searched against the *Bos taurus* (bovine) database, downloaded as a FASTA file from UniProt (UP000009136, 37,501 protein entries, downloaded March 2024), and supplemented with common contaminants (using the built-in option). Trypsin/P was specified as the proteolytic enzyme, and carbamidomethylated cysteine was set as a fixed modification. Oxidation of methionine and acetylation at the protein N-terminus were defined as variable modifications. Default MaxQuant settings were applied for all other parameters, including a precursor mass tolerance (first search) of 20 ppm, fragment ion mass tolerance of 20 ppm, maximum of two missed cleavages, minimum peptide length of seven amino acids, “match between runs” option was disabled, second peptide for identification was enabled, a 1% false discovery rate (FDR) at both the peptide-spectrum match (PSM) and protein level using a target–decoy strategy with reversed protein sequences. Label-free protein quantification (LFQ) was enabled with a minimum ratio count of two^31^. Protein quantification was based on unique and razor peptides, including peptides containing methionine oxidation and protein N-terminal acetylation^31^.

## Data Records

The raw mass spectrometry proteomics data have been deposited in the PRIDE^32,33^ repository archive under project identifier PXD069851^34^. A total of 84 data files were uploaded for this project, comprising 80 RAW format files, 2 MaxQuant related files, 1 FASTA annotation related file and 1 file containing the experimental design.

## Technical Validation

### Step-wise Data Processing and Quality Evaluation for Label-Free Quantification

**Fig. 2:**
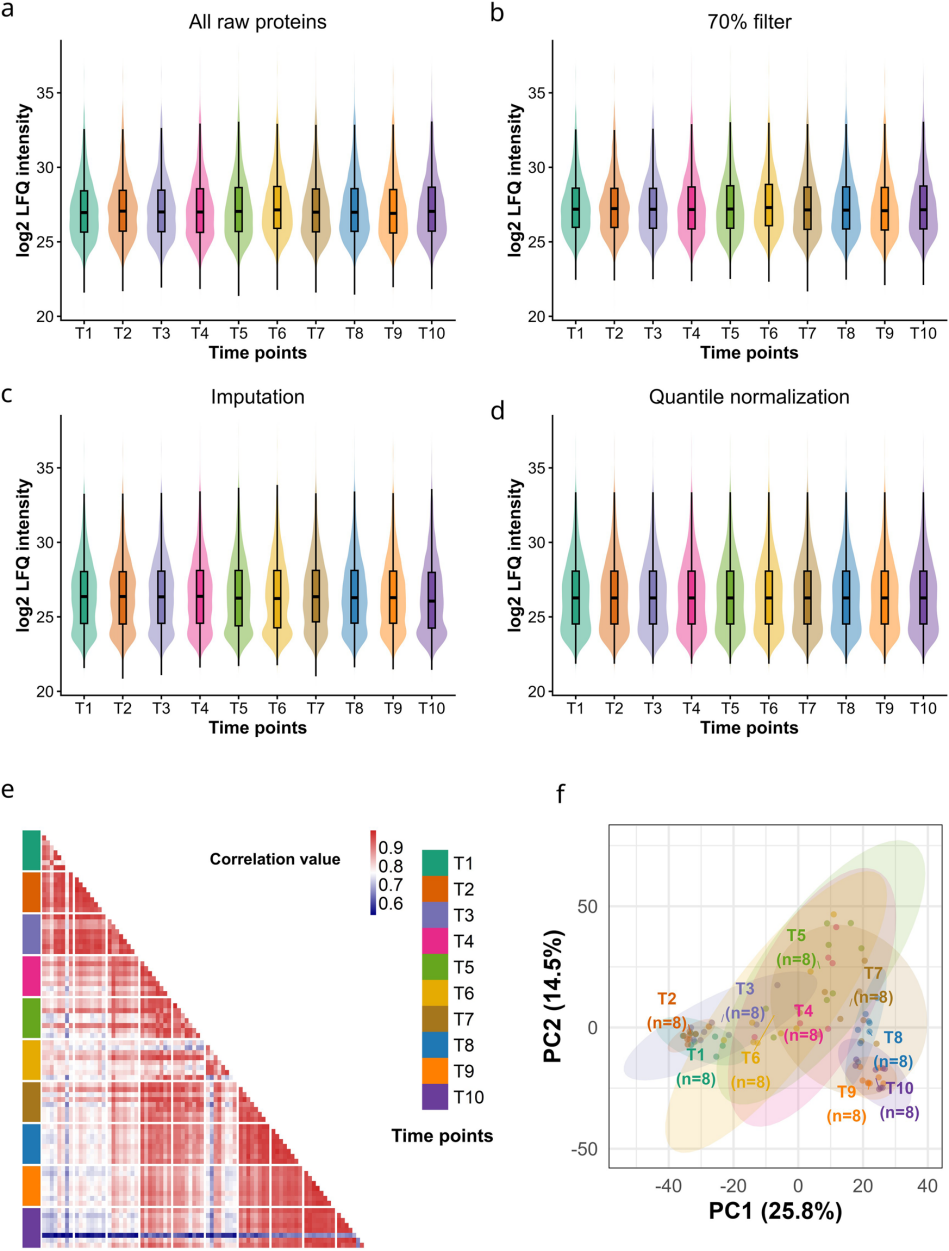
Reproducibility within biological replicates. **(a)** Distribution of label-free quantification (LFQ) intensities of raw data, **(b)** after initial data filtration, **(c)** imputation using a Perseus-style Gaussian imputation strategy, **(d)** and quantile normalization. **(e)** Correlation plot showing the relationships between biological replicates. (**f**) Principal component analysis (PCA) of the normalized proteomic dataset. Samples are projected onto the first two principal components, PC1 and PC2, which explain 25.8% and 14.5% of the total variance, respectively. Confidence ellipses for each time point are overlaid to illustrate variance structure, highlight group separation, and assess reproducibility and clustering of samples based on proteomic profiles.

Label-free quantification (LFQ) intensities generated by MaxQuant were processed in RStudio (2025.05.0 + 496)^35^ using R 4.5.1^36^ with clValid 0.7, openxlsx 4.2.8.1, and base R. Protein groups annotated by MaxQuant as Only identified by site, Reverse, or Potential contaminant were excluded, and proteins were further filtered to retain only those supported by more than one peptide (Peptides > 1), ensuring robust identification. We assessed the total number of proteins identified in each sample to ensure comprehensive coverage of the CL proteome (Fig. 1b–d). In total we identified 3,783 distinct proteins across all groups. LFQ intensity columns were automatically detected using a regular expression (^LFQ intensity\\s), converted to numeric values after removal of thousands separators, and zero or negative intensities were set to missing (NA) prior to transformation. Intensities were then log₂-transformed, and any resulting non-finite values (Inf or NaN) were set to missing to avoid downstream artifacts (Fig. 2a). Sample identifiers were standardized by converting the original Roman-numeral MaxQuant naming convention to a concise timepoint–replicate format (T1_1–T10_8) using a fixed I–X → 1–10 mapping; LFQ sample columns were subsequently recognized using the pattern ^T\\d + _\\d + $, enabling structured, group-aware processing. To balance proteome coverage with data completeness, proteins were filtered using a group-wise 70% validity criterion, whereby a protein was retained if it contained at least 70% non-missing LFQ values within at least one of the ten timepoint groups (T1–T10); with eight biological replicates per timepoint, this corresponded to a minimum requirement of six valid values within a group (Fig. 2b). Quantitative reproducibility across samples was assessed using Pearson correlation coefficients calculated with pairwise complete observations on the log₂ LFQ matrix, and mean correlation values per sample were computed after excluding self-correlations (values < 0.999999); a reference threshold of r = 0.60 was used for quality evaluation, and optional automated filtering of low-correlation samples was applied at mean r < 0.60, subject to a safety constraint requiring at least three valid comparison partners. Protein abundance variability was further quantified as coefficients of variation (CV%) on the linear intensity scale after back-transformation (2^(log₂ LFQ)), calculated both across timepoint means (replicates averaged within each timepoint prior to CV calculation) and within individual timepoints across replicates, providing complementary measures of temporal and within-group variability. Missing LFQ values were imputed using a Perseus-style Gaussian imputation strategy based on the global distribution of observed intensities across all samples (Fig. 2c), with missing entries replaced by random draws from Normal(μ − 1.8σ, (0.3σ)²) (downshift 1.8, width 0.3), and imputation reproducibility ensured by a fixed random seed (set.seed(1))^37^. Following imputation, the LFQ matrix was quantile-normalized using a rank-based procedure (ties.method = “min”) in which each sample’s ordered intensities were replaced by the mean value at the corresponding quantile across all samples, thereby enforcing identical empirical distributions and minimizing technical variability (Fig. 2d). The cumulative effects of filtering, imputation, and normalization were evaluated using sample-wise distribution plots displaying all protein values alongside median ± standard deviation overlays on a shared y-axis, enabling direct visual comparison of distributional stability across processing stages.

### Data reproducibility and consistency

Reproducibility of LFQ intensities across samples was assessed in R 4.5.1 using dplyr 1.1.4, tidyr 1.3.2, ggplot2 4.0.1, patchwork 1.3.2, pheatmap 1.0.13, grid 4.5.1, and scales 1.4.0. All QC was performed on the log₂ LFQ matrix after preprocessing (removal of “only identified by site”, reverse hits, and contaminants; Peptides > 1; renaming samples to T1_1–T10_8; and retaining proteins with ≥ 70% valid values within at least one timepoint, i.e., ≥6/8 replicates). Distribution stability across processing stages was examined using sensitivity plots (RAW log₂ LFQ → 70% filtered → imputed → quantile-normalized) that displayed all protein values per sample (jittered points) with a median ± SD overlay and shared y-axis limits. Correlation QC used Pearson correlation (pairwise complete observations) across all LFQ samples, followed by mean correlation per sample (excluding self-correlations, r < 0.999999) and visualization as an ordered barplot; optional sample exclusion flagged LFQ columns with mean r < 0.60 with a safeguard requiring at least three valid comparison partners before removal (Fig. 2e).

Protein-level variability was quantified as CV% on the linear scale by back-transforming intensities (2^(log₂ LFQ)) and computing (i) an overall CV across timepoint means (replicates averaged within each timepoint before CV) and (ii) replicate CVs within each timepoint (T1–T10). Multivariate structure was evaluated by sPLS-DA using mixOmics 6.34.0 on the quantile-normalized log₂ LFQ matrix organized as samples × proteins with group labels encoded as a T1–T10 factor; model tuning used tune.splsda with M-fold CV (5 folds, 10 repeats), centroids.dist, and BER, testing keepX = 25, 50, 100, fixing ncomp = 9, and ensuring reproducibility with set.seed(1). The final model was visualized as 2D component-pair plots with group centroids and 95% confidence ellipses computed from the empirical covariance and scaled by the χ² radius (df = 2, level = 0.95). In parallel, PCA was performed using mixOmics::pca (center = TRUE, scale = TRUE; up to 15 PCs; set.seed(1)), selecting the minimum number of PCs achieving ≥90% cumulative explained variance (elbow/cumulative criterion) and plotting all 2D PC comparisons with 95% ellipses and centroid labels to confirm replicate tightness and timepoint separation (Fig. 2e).

### Protein differential expression and enrichment analysis

**Fig. 3:**
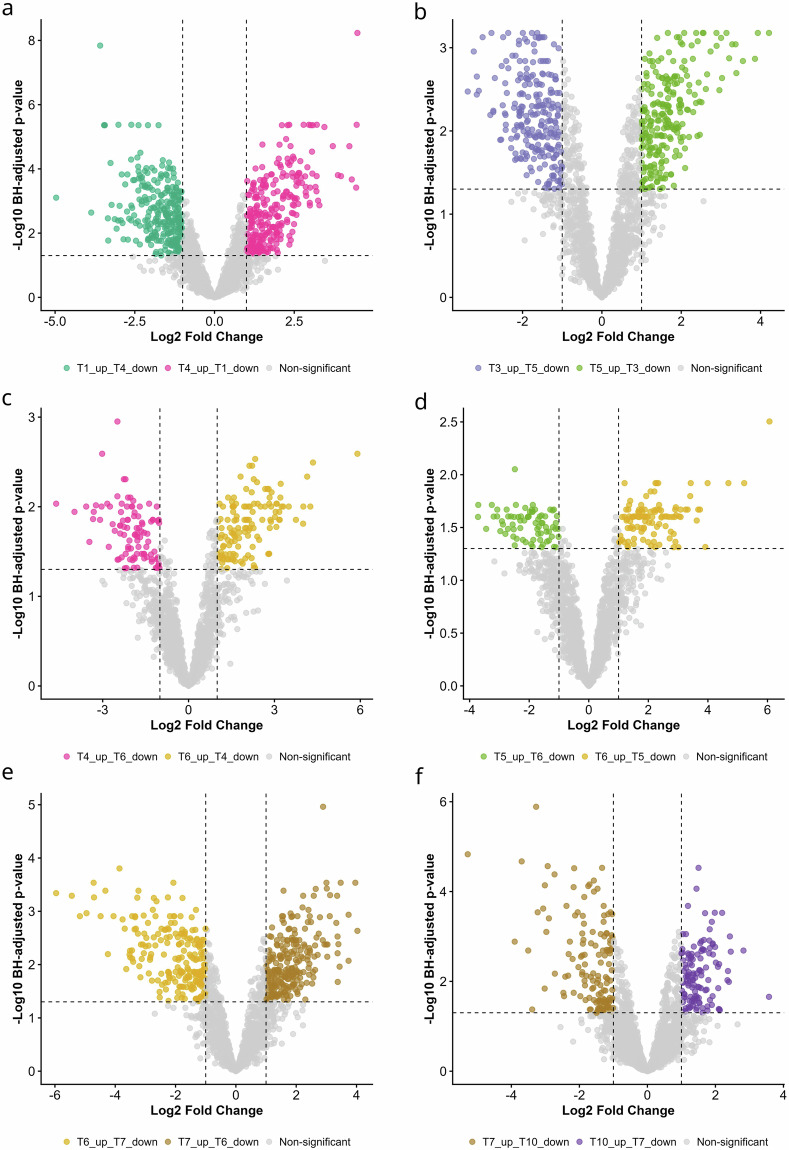
Differential protein expression across corpus luteum phases. Volcano plots illustrate differential protein expression between selected stages of the corpus luteum. Pairwise comparisons were performed between: (**a**) T1 (Days 1–2) vs. T4 (Days 8–12), (**b**) T3 (Days 5–7) vs. T5 (Days 13–17), (**c**) T4 (Days 8–12) vs. T6 (Day > 18), (**d**) T5 (Days 13–17) vs. T6 (Day > 18), (**e**) T6 (Day > 18) vs. T7 (Months 1–2), and (**f**) T7 (Months 1–2) vs. T10 (Month > 7). Each dot represents an individual protein. Differential expression analysis was conducted using the limma framework, with p-values adjusted for multiple testing using the Benjamini–Hochberg false discovery rate (FDR) method. Proteins with adjusted p < 0.05 and |log₂ fold change| > 1 (≥2-fold change) were considered significantly differentially expressed and are highlighted in the corresponding group colors. The y-axis represents −log₁₀(FDR).

**Fig. 4:**
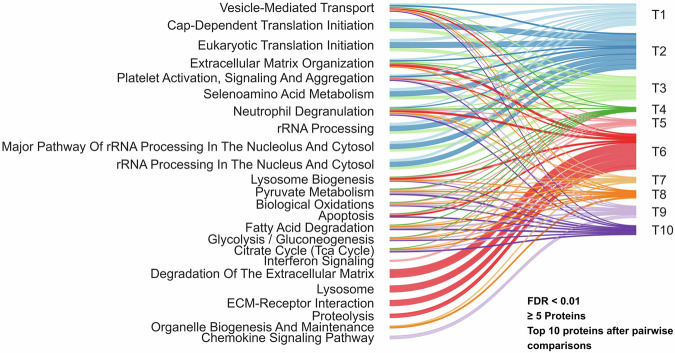
Sankey diagram of pathway enrichment across all time-point comparisons. Sankey diagram depicting links between sample collection time points (right) and enriched biological pathways (left) derived from all pairwise temporal comparisons. Differentially expressed proteins were identified from volcano plot analyses, stratified by up- and down-regulation, and annotated for pathway enrichment using Fisher’s exact test. For each time point, up to TOP_N = 10 pathways meeting FDR < 0.01 and containing ≥ 5 proteins were retained after pathway-key deduplication and keyword filtering. Time points are displayed as ten distinct groups, each represented by a unique color.

Differential abundance across reproductive stages was tested across all pairwise timepoint comparisons (T1–T10) using limma 3.66.0 on the selected expression source (default: quantile-normalized log₂ LFQ matrix; optionally: imputed matrix). For each comparison, a two-group design (~0 + group) was fitted, the contrast was defined as (late − early), and protein-wise inference used moderated t-statistics with eBayes(robust = TRUE); results included log₂FC, moderated t, raw p-values, and Benjamini–Hochberg FDR (BH). Volcano classification used BH-adjusted p < 0.05 and |log₂FC| > 1 (≥2-fold), with −log10(FDR) plotted on the y-axis and dashed reference lines at the FDR and fold-change thresholds. Distinct proteomic patterns were observed across reproductive stages: 282 proteins were upregulated in T1, compared with 268 in T4 (Fig. 3a); T3 versus T5 showed 240 proteins upregulated in T3 and 240 in T5 (Fig. 3b); T4 versus T6 had 98 higher in T4 and 131 in T6 (Fig. 3c); T5 versus T6 revealed 74 elevated in T5 and 109 in T6 (Fig. 3d); cyclic versus pregnant CL (T6 vs T7) exhibited 219 upregulated in T6 and 240 in T7 (Fig. 3e); and within pregnancy, T7 versus T10 showed 138 upregulated in T7 and 125 in T10 (Fig. 3f). Protein identifiers used Gene_name where available, otherwise falling back to Protein IDs, with multi-gene strings normalized by splitting on delimiters and collapsing unique entries to maintain consistent mapping. Functional annotation of significant gene sets used org.Bt.eg.db 3.22.0 and AnnotationDbi 1.72.0 for bovine SYMBOL/ENTREZ mapping, GO.db 3.22.0 for GO term names and ontology, KEGGREST 1.50.0 for bovine KEGG pathway membership (with cached pathway descriptions), and ReactomePA 1.54.0 for Reactome assignment via a stable orthology workflow implemented with biomaRt 2.66.0 (Ensembl archive host), mapping bovine symbols to human Entrez IDs and projecting Reactome pathways back to bovine genes. Over-representation testing used Fisher’s exact tests against a background defined as all genes detected in the processed dataset, with BH correction and an enrichment factor calculated as the ratio of significant-set membership to background membership. Enrichment visualization combined GO/KEGG/Reactome evidence into heatmaps using ggplot2 4.0.1, viridisLite 0.4.3, and scales 1.4.0, applying filters of adjusted p < 0.01 and ≥5 proteins, optional keyword-based pathway focusing, paging (≤15 pathways/page), and gene-label readability controls (≤60 genes/page, auto font/angle/figure size). All timepoints were used for Sankey and resolved functional dynamics were summarized with Sankey diagrams using networkD3 0.4.1, htmlwidgets 1.6.4, RColorBrewer 1.1.3, jsonlite 2.0.0, and webshot2 0.1.2, where pathway selection was performed timepoint-specifically, retaining up to TOP_N = 10 pathways per timepoint meeting FDR < 0.01 and ≥5 proteins (after pathway-key deduplication and keyword filtering) to ensure the Sankey reflected dominant, stage-specific enrichment rather than pathways carried forward only by recurrence across comparisons (Fig. 4).

## Data Availability

The mass spectrometry proteomics data have been deposited in the PRIDE Archive under accession number PXD069851.

## Code availability

The used R code and raw ProteinGroups data from the MaxQuant search have been uploaded to GitHub. This R script is available on GitHub (10.5281/zenodo.18680100).
